# Genomic and Phylogenetic Characterization of *Rhodopseudomonas infernalis* sp. nov., Isolated from the Hell Creek Watershed (Nebraska)

**DOI:** 10.3390/microorganisms10102024

**Published:** 2022-10-13

**Authors:** Christine E. Humphrey, Nicole Burnett, Shivangi Dubey, John A. Kyndt

**Affiliations:** College of Science and Technology, Bellevue University, Bellevue, NE 68005, USA

**Keywords:** *Rhodopseudomonas*, purple non-sulfur bacteria, whole genome, taxonomy, Hell Creek, antibiotic resistance, aromatic compound degradation

## Abstract

The genus *Rhodopseudomonas* is known for its versatile metabolic capabilities and has been proposed to be used in a wide variety of innovative applications, ranging from biohydrogen and electricity production, bioremediation and as biostimulant in agriculture. Here, we report the isolation, characterization and genome sequence analysis of a novel *Rhodopseudomonas* species, strain HC1, isolated from the Hell Creek urban native restoration area. Whole genome-based analysis, average nucleotide identity (ANI) comparison, and growth characteristics identified this isolate as a new species of the *Rhodopseudomonas* genus, for which we propose the name *Rhodopseudomonas infernalis* sp. nov. Besides containing several nitrogenases for nitrogen fixation and hydrogen production, the HC1 genome encodes a unique gene cluster, not found in any other *Rhodopseudomonas* species, which encodes genes for the degradation of yet-unidentified aromatic PCB-type chemicals with potentially interesting biotechnological applications. The genomic features of *Rps. infernalis* HC1 indicate that it plays a positive role in the degradation of anthropogenic substances and aids the restoration of the Hell Creek watershed by contributing to N_2_ and carbon fixation and plant growth; however, the genome also contains several antibiotic resistance genes, indicating a broad range of antibiotic resistance in this environmental isolate.

## 1. Introduction

The Hell Creek watershed area is part of a nature restoration project around Hell Creek, situated in the suburban Omaha area. A collection pond has been constructed that collects rainwater and overflow runoff from a larger commercial and residential development area nearby, but is not directly connected to the creek itself. We had previously performed a metagenomic analysis of the watershed [1] and now enriched a new strain from the HC1 collected samples. The HC1 collection site was part of the pond, located on the north side of the Hell Creek watershed [1]. The metagenomic data showed only a small representation of *Bradyrhizobiaceae* [1]; however, we were able to enrich a *Rhodopseudomonas* isolate by selective cultivation in the lab. Besides the genotypic and phenotypic characterization of the new isolate, we also sequenced its whole genome and performed a whole genome-based taxonomic analysis. These analyses showed that the HC1 isolate should be classified as a new species of the genus *Rhodopseudomonas*.

*Rhodopseudomonas* has been isolated from a wide variety of environments, ranging from aquatic sediments [2,3], sludge [4,5], soils [6,7], alkaline waters [8] and eutrophic ponds [9]. The genus belongs to the family *Bradyrhizobiaceae* and order *Rhizobiales* within the Alphaproteobacteria class [10]. All *Rhodopseudomonas* are Gram-negative, anoxygenic phototrophic bacteria and are characterized by a budding cell morphology and the presence of intracytoplasmic membrane system of the laminar type [10]. *Rhodopseudomonas* belongs to the purple non-sulfur bacteria and grows anaerobically in the light or aerobically in the dark with a variety of different carbon sources and electron donors. However, *Rhodopseudomonas* has the exceptional ability to grow using the four major modes of metabolism, photoautotrophic, photoheterotrophic, chemoautotrophic and chemoheterotrophic, and is flexible in switching between these modes depending on environmental conditions [11,12]. This wide metabolic flexibility has contributed to becoming a model organism for studies on photophosphorylation and light harvesting mechanisms [12,13,14,15]. Its exceptional ability to adapt to changing light conditions, aided by phytochromes that regulate gene expression, has also raised interest in studying *Rhodopseudomonas* to optimize strategies for solar energy capture [16].

All *Rhodopseudomonas* perform nitrogen fixation using nitrogenase enzymes that convert atmospheric N_2_ gas into ammonia (NH_3_) and hydrogen (H_2_), thereby converting the nitrogen into a useable form for bacterial and plant biosynthesis. This same enzyme also provides *Rhodopseudomonas* the capability to produce large amounts of hydrogen (driven by ATP from light energy). *Rhodopdseudomonas* strains have also been studied extensively for their ability to degrade a wide variety of organic compounds [17,18,19]. Several strains dehalogenate and degrade chlorinated benzoates and other aromatic compounds often found in industrial wastes [11,12,19].

Due to its metabolic diversity, the ability to capture N_2_ and produce hydrogen using light energy, and its ability of anaerobic degradation of aromatic compounds, *Rhodopseudomonas* has been proposed as an ideal bacterium for a variety of biotechnological applications, ranging from environmental bioremediation and biostimulation, to biofuel (biohydrogen) production and electricity production [12,13,20,21].

*Rhodopseudomonas palustris* was initially identified by van Niel in 1944 [22]. There are currently 7 validly named species in the *Rhodopseudomonas* genus: *Rps. palustris*, *Rps. rutila*, *Rps. faecalis*, *Rps. pantothenatexigens*, *Rps. thermotolerans*, *Rps. rhenobascensis*, and *Rps. telluris* [23,24,25]. Several genomes of different strains of *Rhodopseudomonas* have been sequenced. As of to date, a total of 28 *Rhodopseudomonas* genomes were found in NCBI Genbank. Eight of them are from strains designated as *Rps. rutila*, two as *Rps. palustris*, four as *Rps. faecalis*, one as *Rps. pantothenatexigens*, *Rps. thermotolerans*, *Rps. rhenobacensis*, “*Rps. boonkerdii*”, and 10 of them have no species designation. With more genomic information becoming available on this genus in the last few years, it became clear that there was a need for taxonomic revision of the genus as several taxonomic discrepancies were described with new genomes being published [3,26]. Since *Rhodopseudomonas* is widely distributed in soil and water environments it is frequently found in metagenomic analyses and correct taxonomic information is important to derive detailed conclusions from such broad metagenomic studies. Therefore, an updated phylogenetic analysis of the genus, based on whole genome comparisons and 16S rRNA comparisons, was published recently [25], and revealed that the genus shows great heterogenicity at the species level. That study also showed that *Rhodopseudomonas palustris* DSM 123 is the authentic type strain for that species, which had previously been debated, but previous confusion was shown to be due to errors in strain transfers over time. It also placed several of the previously misplaced strains into the *Rps. rutila* species group and confirmed *Rps. rutila* R1 as the type strain.

A whole-genome-based comparison was performed with the new isolate HC1, and found the new isolate to be only distantly related to any of the currently described species. We now present the complete genomic and physiological characterization of this new species, for which we propose the name *Rhodopseudomonas infernalis* sp. nov.

## 2. Materials and Methods

### 2.1. Bacterial Isolation and Cultivation

Microbial samples were isolated from Hell Creek in Omaha, Nebraska in April 2020, as described in Kyndt, 2020 [1]. Samples of 50 mL were collected from the top 30 cm of the water surface using sterile collection tubes and immediately transferred to the lab where they were stored overnight at 4 °C. The next day 100 μL of sample HC1 was plated on RCV media [27], supplemented with biotin (15 μg/L) and nicotinic acid (1 mg/L) and grown anaerobically at 35 °C for at least two weeks until colonies appeared. Pink-red colonies were used to streak fresh RCVBN agar plates and pure cultures were obtained after three rounds of dilution plating. Purified cultures were scaled up in 250 mL flasks with RCVBN media, filled to the top and closed off anaerobically. p-Amino benzoic acid (pABA) was added to the growth media at a concentration of 2 mg/L. For the growth with various carbon/electron sources, we replaced the malic acid in the RCVBN media with equimolar amounts (30 mM) of the respective carbon source. Each of the growth media was tested both under anaerobic light (full spectrum light at 32 °C in an anaerobic jar with EZ GasPak from Becton Dickinson, NJ, USA) or aerobic dark conditions (35 °C dark incubator). Growth of the new isolate was tested on RCVBN media agar plates under both aerobic dark and anaerobic light conditions with the following carbon sources: malate, citrate, fumarate, L-aspartate, L-glutamate, L-arginine, tartrate, D-gluconate, propionate, benzoate, D-glucose, D-fructose, and casamino acids. Plates were grown aerobically and incubated at 35 °C in the dark and inspected for growth after two weeks of incubation. Plates grown anaerobically in the light at 30 °C were inspected after at least one month of incubation. A second transfer on each respective media was performed before counting positive growth. For antibiotic growth studies, ampicillin (Sigma-Aldrich, St. Louis, MO, USA) was added to the RCVB media plates (with fumarate) to a final concentration of 100 μg/mL and incubated under aerobic conditions in the dark. The growth pH range was investigated between pH 5 and 11 in liquid anaerobic cultures (RCVBN media) in the light. Absorption spectra of whole cell (from anaerobic light growth) were obtained by diluting the cells with glycerol to decrease scattering (1:1 ratio), using an Evolution 300 UV-vis spectrophotometer (Thermo Scientific, Waltham, MA, USA) with the VisionPro software (version 4.5.0). Pigment extraction was performed by centrifugation of 1 mL of stationary phase culture (5 min at 10,000× *g*) and resuspending the pellet in 1 mL of a methanol/acetone mixture (50/50). The suspension was sonicated for 2 min (10 s pulses, 70% amplitude) using a microtip model FB120 Sonicator (Thermo Scientific) and centrifuged for 5 min at 5000× *g*. Spectra were collected from the supernatans using a Genesys 10S UV-vis spectrophotometer (Thermo Scientific).

Cells were observed using a MEIJI Techno (Chikumazawa, Japan) MT4200H microscope equipped with brightfield and phase contrast condenser, and images were obtained with an attached Motic digital camera (Motic North America, Richmond, BC, Canada).

### 2.2. DNA Purification

DNA was isolated from bacterial colonies grown under anaerobic light conditions, using the GeneJet DNA purification kit (Thermo Scientific). Purified DNA sample was analyzed for purity and concentration using a Nanodrop and Qubit, showing an absorbance 260/280 nm ratio of 1.7 with a concentration of 31.5 ng/µL. An amount of 300 ng purified gDNA was used for Illumina whole genome sequencing, and 1 μg was used for Oxford Nanopore long read sequencing.

### 2.3. Next Generation Sequencing

The Illumina DNA library was prepared following the Nextera DNA Flex library prep kit (Illumina, San Diego, CA, USA). Genomes were sequenced using 500 μL of a 1.8 pM library with an Illumina MiniSeq instrument. Paired-end (2 × 150 bp) sequencing generated 1,842,158 reads and 278.2 Mbps. Quality control of the reads was performed using FASTQC within BaseSpace (Illumina, version 1.0.0), using a kmer size of 5 and contamination filtering. The data was assembled de novo using Unicycler [28] within PATRIC (now BV-BRC) [29]. This assembly yielded 87 contigs (>300 bp), with an N50 of 113,479 bp. Oxford Nanopore DNA library prep was performed following the Ligation Sequencing Kit (SQK-LSK110) on a FLO-MIN106D flow cell with a MinION-Mk1B instrument [30]. Read QC and fast basecalling were performed using Guppy [31] within the MiniKnow software. We used 40,000 reads of this long-read sequencing dataset which yielded 219 Mbps of sequencing data with an average read length of 5475 bp. A combined de novo genome assembly with the Illumina high fidelity sequencing was performed using Unicycler within PATRIC (now BV-BRC). This improved the contig number to 43 contigs, and increased the N50 to 275,454 bp. In both cases, the coarse and fine consistency were >98% and >96%, respectively. The final assembled genome was found to be 99.3% complete with less than 1% contamination. This WGS has been deposited to NCBI Genbank with accession number NZ_JAJNAL000000000.

The genome sequence was annotated using RAST (Rapid Annotations using Subsystem Technology; version 2.0; [32]. Taxonomic classification of the assembled contigs was performed with Kraken2 within PATRIC (now BV-BRC) [33]. All database genomes were used for this taxonomic classification.

### 2.4. Whole Genome Comparison

Average percentage nucleotide identity (ANIb) between whole genomes was calculated using JSpecies [34], which uses a pairwise genome comparison algorithm to measure probability if two or more genomes belong to the same species. A whole genome-based phylogenetic tree was generated using the CodonTree method within PATRIC [29]. CodonTree used the amino acid and nucleotide sequences from a defined number of PATRIC’s global Protein Families (PGFams) to build an alignment, and then generated a tree based on the differences within those selected sequences. Both the protein (amino acid) and gene (nucleotide) sequences were used for each of the selected genes from the PGFams. The aligned proteins and coding DNA from single-copy genes were used for RAxML analysis [35,36]. Protein sequences were aligned using MUSCLE [37], and the nucleotide coding gene sequences were aligned using the Codon_align function of BioPython. A concatenated alignment of all proteins and nucleotides were written to a phylip formatted file, from which a partitions file for RaxML was generated. The support values for the phylogenetic tree are generated using 100 rounds of the ‘Rapid bootstrapping’ option of RaxML. iTOL was used for tree visualization [38].

The single protein tree for protW, was generated by the multiple sequence alignment viewer within PATRIC [29], which uses MUSCLE [37] for alignment and FastTree [39] for tree design. The Newick files were downloaded to iTOL [38] for visualization.

### 2.5. 16S rDNA Phylogenetic Comparison

The multiple sequence alignment for the 16S rRNA comparisons was performed using ClustalW [40]. The evolutionary history was inferred by using the Maximum Likelihood method and Hasegawa-Kishino-Yano model [41]. The bootstrap consensus tree inferred from 500 replicates was taken to represent the evolutionary history of the taxa analyzed [42]. The percentage of replicate trees in which the associated taxa clustered together in the bootstrap test (500 replicates) are shown next to the branches. Initial tree(s) for the heuristic search were obtained automatically by applying the Maximum Parsimony method. A discrete Gamma distribution was used to model evolutionary rate differences among sites (5 categories (+G, parameter = 0.2113)). The rate variation model allowed for some sites to be evolutionarily invariable ([+I], 73.59% sites). This analysis involved 38 nucleotide sequences that were either downloaded from Genbank or obtained from the genome sequences where available. All positions with less than 95% site coverage were eliminated, i.e., fewer than 5% alignment gaps, missing data, and ambiguous bases were allowed at any position (partial deletion option). There were a total of 1362 positions in the final dataset. Evolutionary analyses were conducted in MEGA X [43].

## 3. Results and Discussion

### 3.1. Morphology and Photopigments

**Figure 1:**
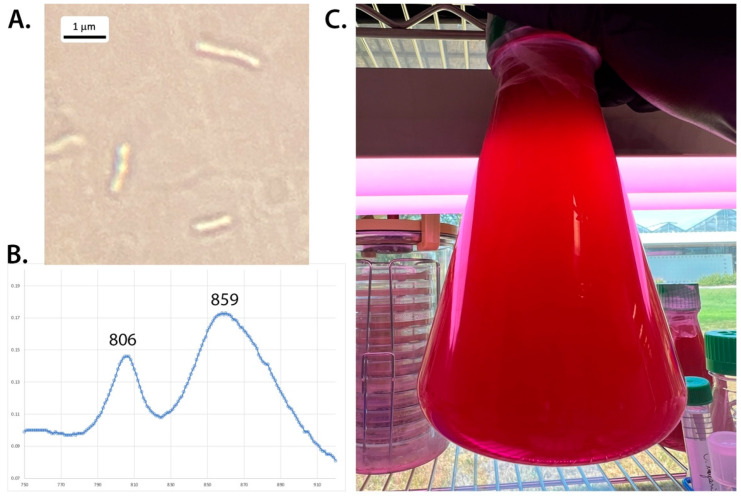
(**A**) Microscopy of *Rhodopseudomonas* strain HC1 at 1000× total magnification. (**B**) Absorption spectra of from whole cells of *Rhodopseudomonas* HC1 shows near IR absorption characteristic of BChl *a*. (**C**) Cultures of *Rhodopseudomonas* HC1 show a distinctive red color.

Environmental water samples were collected from the northern pond at the Hell Creek watershed in 2020 as described earlier [1]. A small amount of sample HC1 (Hell creek location 1) was used to inoculate RCVBN media plates. Strain HC1 appeared as pink colonies on RCVBN plates grown photosynthetically at 32 °C after about 3 weeks of incubation. Initial isolates appeared to have a fast-growing colorless contaminant; however, after three transfers on anaerobic RCVBN plates, the red-pink colonies were the only colonies observed on the plates and were used to inoculate liquid RCVB cultures and were considered pure cultures. Upon microscopic examination, isolate HC1 consisted of rod-shaped cells (0.2–0.5 um wide and 1.2–2 um long) (Figure 1). Cells were found to be motile. Older cells appeared to have a swelling at the polar ends, giving them a slight dumbbell shape appearance. Whole cell absorption spectra of strain HC1 showed maxima at 806 nm, 859 nm (and a broad shoulder at 884 nm) (Figure 1), indicative of bacteriochlorophyll *a*. Pigment extraction with MetOH/acetone revealed additional spectral peaks with maxima of 368 nm (with a 394 nm small shoulder), and broad maxima at 498, 533, and 589 nm, indicative of the presence of spirilloxanthin-type carotenoids, as found in other *Rhodopseudomonas* species [10,44,45].

### 3.2. Physiological Characteristics

Strain HC1 was able to grow anaerobically in the light and aerobically in the dark; however, anaerobic light growth was very slow (color appeared after about 2–3 months of incubation). This anaerobic photosynthetic growth of the purified HC1 strain appears to be considerably slower than other *Rhodopseudomonas* species, and chemotrophic growth appears to be preferred in this strain [10]. The following organic compounds were found to be used as carbon source: malate, tartrate, D-gluconate, fumarate, L-aspartate, L-glutamate, casamino acids, L-arginine, and D-glucose (Table 1). Interestingly, of all the other *Rhodopseudomonas* strains tested, none of them were able to use L-arginine as the sole carbon source for heterotrophic growth [10,23,24]. Although we found strain HC1 to grow well under aerobic dark conditions on L-glutamate, anaerobic phototrophic growth on this substrate was very slow compared to other carbon sources. No growth occurred on citrate, propionate, benzoate and fructose. Growth occurred at 25, 35 and 45 °C, but was found optimal at 35 °C. Strain HC1 was capable of growth from pH 6 to 10 in anaerobic cultures in the light; however, it grew optimally at pH 7–7.5. Growth was inhibited by > 1% NaCl.

### 3.3. Genome Sequence

To identify the taxonomy of the isolated strain, we decided to sequence its whole genome. The whole genome sequencing provided a genome assembly with a genome length of 5,543,543 bp, in 43 contigs, and an average G+C content of 65.3%. The latter is close to the GC content of several of the other *Rhodopseudomonas* species [10,23,24]. An initial taxonomic classification of these assembled contigs using Kraken2 confirmed that this genome belongs to *Rhodopseudomonas* (68% of the identified reads). About 31% resulted in no hits from the database, which indicates that this is a species or strain that is not represented in the database.

### 3.4. Whole Genome-Based Phylogenetic Analysis

Since there appeared to be much heterogeneity within the *Rhodopsuedomonas* genus [25], we decided to perform a more in-depth whole genome comparison of this new isolate to obtain a more complete phylogenetic analysis. Average percentage nucleotide identity (ANIb) between our genome and the whole genomes of other *Rhodopseudomonas* species was calculated using JSpecies (Table 2) [34]. Bacteria with ANI > 97% subjectively have only a few nucleotide differences and virtually no amino acid substitutions or insertions or deletions of either genes or amino acid residues. There would be no argument as to whether they were members of the same species. Bacteria with ANI of 95–97% will have minimal amino acid substitutions and virtually no insertions or deletions and would also be recognized as same species [34].

Bacteria with ANI < 90% will have insertions and deletions of whole genes, they will have substantial base changes, and most proteins will have at least a few amino acid substitutions. In other words, they would be recognized in most cases as separate species. As can be seen in Table 2, all of the *Rhodopseudomonas* strains show ANI values < 90% to the new strain HC1. The strain is closely related to *Rps.* ATH 2.1.18 and *Rps.* AAP120, but with only 86.9–87.9% ANI. Strain HC1 is only distantly related to the type strains *Rps. palustris* DSM 123 (83.1% ANI), *Rps. rutila* R1 (81.6% ANI), *Rps. faecalis* JCM11668 (77.3% ANI), *Rps. thermotolerans* JA576 (82% ANI), and *Rps. rhenobascensis* (78.6% ANI). Therefore, strain HC1 should be regarded as a new species, different from the previously characterized *Rhodopseudomonas* species.

**Figure 2:**
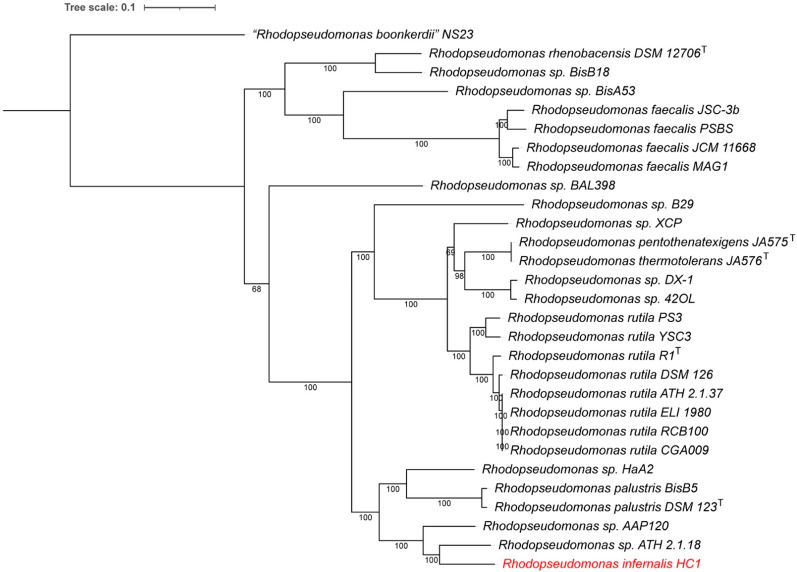
Whole genome-based phylogenetic tree of all sequenced *Rhodopseudomonas* strains. The phylogenetic tree was generated using the codon tree method within PATRIC. The tree is rooted at midpoint. The *Rhodopseudomonas* isolate from Hell Creek (HC1) is indicated in red.

A whole genome-based phylogenetic analysis of the *Rhodopseudomonas* genomes, is consistent with the ANI analysis presented above (Figure 2). The phylogenetic tree was generated using whole genome comparison, with 713 aligned proteins from single-copy genes. As can be seen in Figure 2, strain HC1 is on a separate clade with strains 2.1.18 and AAP120. The closest named species is *Rps. palustris* (strains DSM 123^T^ and BisB5); however, these are on a separate clade and only have low ANI comparison values, indicating that HC1 belongs to a different species. The species *rutila*, *faecalis*, *pentothenatexigens* and *thermotolerans* are clearly more distantly separated on the whole genome tree, which is consistent with our earlier overview of the *Rhodospeudomonas* genus [25].

**Figure 3:**
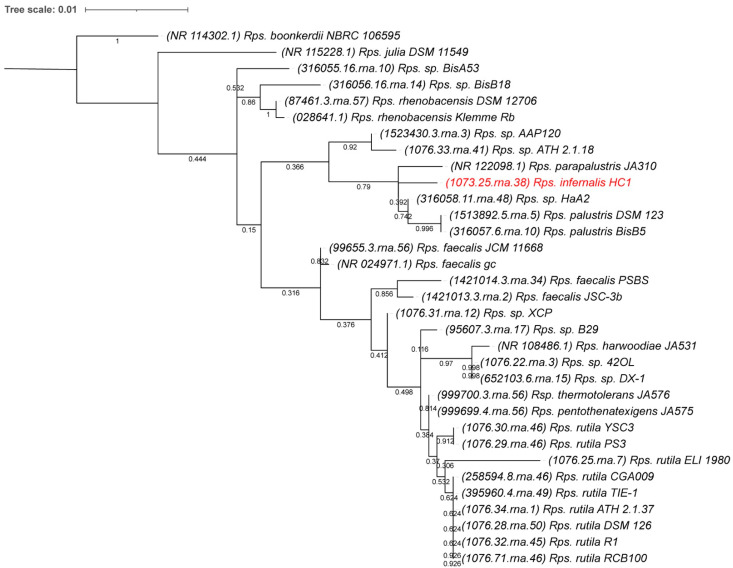
16S rRNA-derived phylogenetic tree for *Rhodopseudomonas* species. The phylogenetic tree was calculated in MegaX and iTOL was used to draw the phylogenetic trees expressed in the Newick phylogenetic tree format. Bootstrap values were generated from 500 bootstrapping rounds. The new isolate, *Rps. infernalis* HC1, 16 rRNA is indicated in red. Accession numbers from either NCBI Genbank or BV-BRC numbers from genome-derived sequences, are indicated in parentheses.

Since several species of *Rhodopseudomonas* do not have genomes available yet, we also performed a 16S rRNA-based phylogenetic comparison to include all species in the comparison. The 16S rRNA sequences used were deducted from the whole genomes where available or directly obtained from NCBI Genbank. The resulting 16S rRNA phylogenetic tree (Figure 3) shows that, based on 16S rRNA comparions alone, the closest relatives to the new isolate are *Rps. parapalustris* JA310 and *Rps.* strain HaA2. No genome is available for strain JA310, but with only 96.2% 16S rRNA identity to the new isolate, this is likely a different species, since this is below the proposed species delineation for 16S rRNA comparisons of 98.7% [46].

The ANI, 16S rRNA and whole genome phylogenetic comparisons all clearly indicate that our newly isolated *Rhodopseudomonas* strain HC1 belongs to a species that is not represented by any of the previously characterized type strains and therefore should be designated as a novel species.

### 3.5. Photosynthetic Gene Cluster

*Rps. infernalis* HC1 contains a conserved cluster of the structural components of the photosynthetic reaction center, a light-harvesting complex 1 (LHC1) system and genes for carotenoid and bacteriochlorophyll synthesis, with a highly conserved synteny compared to the other *Rhodopseudomonas* genomes. Similar to other *Rhodospseudomonas* genomes, the HC1 genome also contains the transcriptional regulators *PpsR1* and *PpsR2* that repress photosystem development under high aeration conditions [47]. The genome also contains at least six annotated bacteriophytochromes, consistent with earlier observations that *Rhodopseudomonas* had developed a unique and sophisticated set of photoreceptors and light responsive regulators to respond to changes in light quality and intensity [14,15,16,48].

The synteny downstream from the photosynthetic gene cluster in strain HC1 is only conserved in strain ATH 2.1.18, which is also the closest relative based on the WGS comparison (Figure 2). Strain 2.1.18 was originally isolated and purified by C.B. van Niel in 1944 from California, and its genome sequenced [49], but unfortunately no formal description of the strain is available.

**Figure 4:**
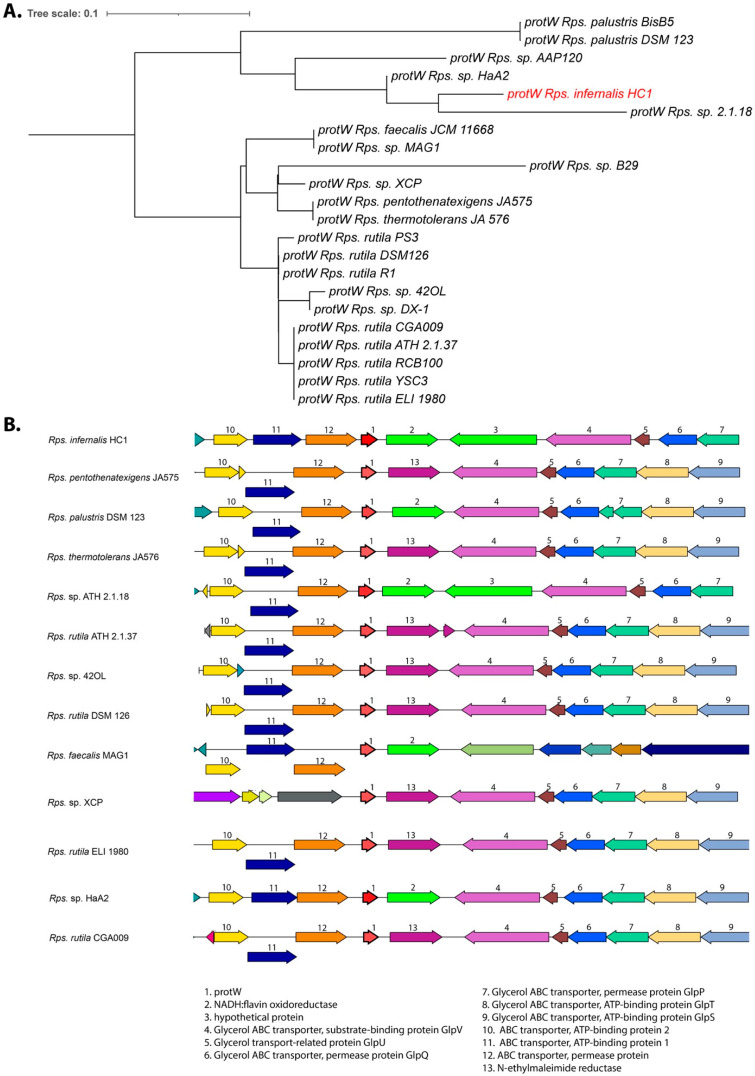
(**A**) Phylogenetic comparison of the protein W amino acid sequences of all sequenced *Rhodopseudomas* species. The tree was rooted at midpoint. Strain HC1 protW is indicated in red. (**B**) Synteny analysis of the protW gene area of *Rhodopseudomonas* species related to isolate HC1. Genes are colored based on their family membership.

*Rhodopseudomonas* is unique amongst the purple nonsulfur bacteria in the fact that the reaction center-LH1 complex can both obtain an open and a closed confirmation [50]. The open conformation has been shown to require the involvement of a small (11 kDa) protein (designated protein W; RPA 4402), which is not found in the photosynthetic gene cluster, but is part of a group of ABC transporter genes elsewhere in the genome. We found the gene for protein W to be present in the genome of strain HC1, and a phylogenetic comparison of this protein also showed strain 2.1.18 as the closest relative (Figure 4A).

The protW gene synteny is conserved in different groups of *Rhodopseudomonas*. Some have protW clustered with an NADH:flavin oxidoreductase (as in HC1) while others have an N-ethylmaleimide reductase (Figure 4B). The main difference, however, is the presence of an additional (614 aa.) hypothetical protein only found downstream of protW in strains HC1 and ATH 2.1.18 (gene 3 in synteny plot in Figure 4B). The presence of protein W indicates that strain HC1 is likely also able to form the open RC-LH1 complex, which is thought to help form a gateway for quinone/quinol exchange [50]. Note that the photosynthetic gene cluster synteny and the protein W phylogeny is also consistent with our earlier taxonomic revision of the genus *Rhodopseudomonas* that confirmed strain DSM 123 as the true *palustris* type strain and the revision of the *Rps. rutila* species group [25].

### 3.6. Growth and Respiration Genes

Strain HC1 contains both forms of ribulose-1,5-bisphosphate carboxylases (RubisCO) for carbon fixation: both genes encoding Form I Cbb large and small subunits, and Form II CbbM larger single RubisCO proteins are present in the genome. The presence of two forms of RubisCO is similar to other *Rhodopseudomonas* strains, and a few other purple nonsulfur bacteria such as *Rhodocyclus* [51,52]. Five carbonic anhydrases (2 alpha, 2 beta and one gamma class) and genes for carbon monoxide utilization were also found.

Genes for cytochrome cbb_3_ oxidase, cytochrome aa_3_ oxidase and a cytochrome bd ubiquinol oxidase are all present in strain HC1, similar to other *Rhodopseudomonas* strains. It also has cytochrome P450 genes for substrate hydroxylation and pyrroloquinoline quinone (PQQ)-dependent periplasmic dehydrogenases. Strain HC1 also has a complete set of *nap*, *nir*, *nor* for denitrification. The *nos* gene cluster (seven genes) that allows growth under anaerobic dark conditions with nitrous oxide as a terminal electron acceptor is missing from strain HC1. Most *Rhodopseudomonas* strains have the *nos* genes, except for strains AAP120, BisB5, B29 and *faecalis* PSBS. Strain HC1 also contains the genes for the low-oxygen sensor regulatory system FixLJ-K, which is present in all *Rhodopseudomonas* and widely distributed in the rhizobiaceae.

Strain HC1 is the only *Rhodopseudomonas* strain tested so far that was able to use L-arginine as a sole carbon source for heterotrophic growth (Table 1). Interestingly, the HC1 genome contains a unique D-amino acid oxidase (EC 1.4.3.3; PGF_00420748) that is one of the first enzymes involved in D-arginine and D-ornithine metabolism. The genome also contains a unique amino-acid racemase encoding gene (PGF_08932911) elsewhere in the genome, that could be involved in D-L-arginine racemization. The unique D-amino acid oxidase gene is surrounded by several unique hypothetical proteins, so further research would be needed to unveil the metabolic pathway involved in arginine-based growth in this strain.

### 3.7. Nitrogen Fixation and Hydrogen Production

As expected for *Rhodopseudomonas*, strain HC1 contains genes for nitrogen fixation and hydrogen production. Several genes were found involved in the assembly, synthesis and regulation of nitrogenases in the HC1 genome. At least 4 molybdenum-iron nitrogenases (alpha and beta chain) genes and one iron-iron nitrogenase (alpha-beta-delta chain) were annotated, besides several *nif* assembly and regulatory proteins. Nitrogenases convert nitrogen gas to ammonia and generate hydrogen gas as part of their catalytic mechanism. The presence of several nitrogenase subunit genes in strain HC1 indicates its ability for nitrogen fixation and hydrogen production, as expected for a *Rhodopseudomonas* species.

### 3.8. Aromatic Compound Degradation

*Rhodopseudomonas* has been recognized for its distinctive ability to degrade aromatic compounds [11,12]. However, there are specific differences between strains in the type of aromatic carbons that can be utilized. Particularly the anaerobic benzoate degradation pathway appears to be lacking from some strains (for example HaA2) but is present in several other strains [2,14]. We found that strain HC1 was unable to grow on benzoate in our minimal medium growth experiments, which sets it apart from the *Rps. palustris* type strain DSM 123 and *Rps rutila* type strain R1, and several other strains. The HC1 genome is indeed also lacking the cluster of genes for the benzoate degradation pathway (RPA0650–RPA0673), similar to the closely related strains AAP120 and 2.1.18, and HaA2.

Interestingly, a closer comparison of unique genes in the *Rps.* strain HC1 genome, identified a cluster of four genes related to aromatic PCB degradation that is only present in this strain. The gene cluster consists of a 4-hydroxyphenylacetate-3-monoxygenase (PGF_00701895) and a catechol-1,2-dioxygenase (E.C.1.13.11.1; PGF_00416715) preceded by two shorter genes encoding a flavin reductase and a putative ester cyclase. 4-hydroxyphenylacetate-3-monoxygenase has been shown to be important in aromatic PCB degradation and attacks a broad spectrum of phenolic compounds. It uses FADH2 as a substrate, produced by a flavin reductase [53], which is likely the function of the upstream gene for flavin reductase in the HC1 genome. Catechol-1,2-dioxygenase is involved in the metabolism of chlorinated or fluorinated benzoate compounds and specifically catalyzes the ring opening to produce cis, cis-muconate derivatives (which are eventually degraded to acetyl-CoA) [54,55,56]. The surrounding sequences of this gene cluster appear to be unrelated to these functions. Downstream of this gene cluster are two small hypothetical proteins (in opposite orientation), followed by several vitamin B12 transporter genes. Upstream of the gene cluster (in opposite orientation) is a gene for a transcriptional regulator (LysR family). The closest relative where this gene cluster is found is *Bradyrhizobium* sp. strain 41S5, which is a soybean nodulating bacteria found in eastern Canada [57], although the substrate for these annotated enzymes was also not identified in the *Bradyrhizobium* genome.

The presence of this unique gene cluster indicates that strain HC1 is capable of the degradation of some specific aromatic PCB-type chemicals with potentially interesting biotechnological applications [55,56]. Further biochemical studies will be needed to identify the nature of these chemicals and exact breakdown pathway.

### 3.9. Antibiotic Resistance

There is a growing global concern with increasing antibiotic resistance and consequently the search for antibiotic resistance signatures is also becoming important in environmental samples [58,59,60]. At least some *Rhodopseudomonas* strains are known to have natural resistance to some antibiotics [11], requiring high concentrations to maintain selective conditions when performing recombination experiments [61,62]. A genome search for antibiotic resistance related genes in the genome of strain HC1 revealed 41 hits from the PATRIC antibiotic resistance feature database. Besides at least 9 multidrug efflux systems (both MdtABC-TolC and TriABC-OmpH systems), the genome also contains a Class D beta-lactamase (EC 3.5.2.6). Based on the presence of these systems the strain is expected to be resistant to beta-lactam (penicillin-type) antibiotics, triclosan, isoniazid and aminocoumarin (novobiocin) antibiotics. Indeed, growth of strain HC1 was tested on RCVB-fumarate plates with ampicillin (100 μg/mL) and growth did occur under dark aerobic conditions. However, further specificity and susceptibility studies will be needed to confirm the full spectrum of antibiotic resistance present. The presence of antibiotic resistance genes in strain HC1 is not surprising given the fact that the Hell Creek pond is supplemented with runoff from a larger area that includes residential and commercial buildings and roads, that likely contain residual amounts of antibacterial pollutants. In fact, a whole genome-based metagenomic analysis of the Hell Creek watershed indicates a substantial amount of gene signatures (2–3% of the total reads) related to resistance to antibiotics and toxic compounds present in the entire microbiome of the catch pond where strain HC1 was isolated (Kyndt, unpublished), consistent with this hypothesis.

### 3.10. Description of Rhodopseudomonas infernalis *sp. nov.*

Based on the genomic, genetic and physiological differences described above, we propose that strain HC1 belongs to a new species and propose the name *Rhodopseudomonas infernalis* sp. nov. for this novel species.

*Rhodopseudomonas infernalis* (N.L. neut. n. *inferno* Hell; *infernalis* isolated from Hell creek).

Cells are rod-shaped, 1–2 mm long, and thin (0.2–0.5 mm wide). Cells appear to widen at the poles, creating a dumbbell shape (Figure 1; similar to *Rps. palustris* [10]). Cells are motile. Color of the cell suspension is red to dark red. Phototrophically grown cells show absorption maxima indicative of bacteriochlorophyll *a* (near IR maxima at 806 and 859). Extracted carotenoids are of the spirilloxanthin type. Photoheterotrophic growth in anaerobic conditions and chemothrophic growth under dark oxic conditions is possible with a variety of organic substrates: malate, tartrate gluconate, fumarate, aspartate, glutamate, casamino acids, arginine, and D-glucose (Table 1). No growth occurred on citrate, propionate, benzoate and L-fructose. Growth occurs at 25, 35 and 45 °C, but is optimal at 35 °C. pH optima is 7.0–7.5 (pH range: 6–10) The genome size is 5.5 Mbp (Genbank accession number NZ_JAJNAL000000000) and DNA G+C content is 65.3 mol%.

Strain HC1 was isolated from pond water at the Hell Creek watershed in Omaha, Nebraska, US.

## 4. Conclusions

A new photosynthetic strain was isolated from the Hell Creek native restoration area in suburban Omaha, NE. The isolate was found to be a new species of *Rhodopseumonas* based on genomic and physiological comparisons, for which we proposed the name *Rhodopseudomonas infernalis* sp. nov. The species showed both phototrophic and chemotrophic growth on a wide range of organic substrates and the genome also contains genes for the degradation of yet unidentified aromatic PCB-type chemicals. The genome also contains several nitrogenases, consistent with *Rhodopseudomonas* ability to fix nitrogen, which stimulates the growth of plants and microbes.

The physiological and genomic features of *Rps. infernalis* HC1 indicate that its presence at the Hell Creek restoration area contributes to N_2_ and carbon fixation and plant growth and plays a positive role in the degradation of anthropogenic substances. However, the genome also contains several antibiotic resistance genes, indicating a broad range of antibiotic resistance in this environmental isolate. The presence of antibiotic resistance genes in new isolates from urban native restoration areas contributes to a growing global problem of wide-spread environmental traces of antibiotic resistance.

## Figures and Tables

**Table 1:** Physiological growth characteristics of strain HC1 as compared to the type strains of *Rps. palustris* DSM 123^T^, *Rps. julia* DSM 11549^T^, and *Rps. rhenobacensis* Rb^T^. +, good growth; -, no growth; (+), weak growth (OD_660_ < 0.2 or absent on plates). Data were from this study, and [10,23].

Characteristic		Strain HC1	*Rps. palustris* DSM123	*Rps. julia* DSM11549	*Rps. rhenobacensis* Rb
cell shape		Rod	Rod	Rod	Rod
Cell width (um)		0.2–0.5	0.8–1.2	1.0–1.5	0.5–0.8
Cell length (um)		1.2–2.0	2.0–3.0	2.5	1.5–2.0
Motility		+	+	+	+
pH optimum		7–7.5	6–9	6–6.5	5–8
G+C content (mol%)		65.3 *	64.1 *	63.5	65.4
Abs. maxima near IR (nm)		806, 859	800, 861–863	803, 850	805, 878
Organic carbon sources:					
	Propionate	-	+	+	-
	Citrate	-	(+)	-	-
	Malate	+	+	+	+
	Tartrate	+	-	-	+
	D-Gluconate	+	+	nd	+
	L-Aspartate	+	-	+	nd
	L-Glutamate	+	-	-	-
	Casamino acids	+	+	nd	nd
	L-Arginine	+	-	nd	-
	Benzoate	-	+	-	-
	D-Glucose	(+)	(+)	+	-
	D-Fructose	-	(+)	+	-
	Fumarate	+	+	+	+
Isolation source		Pond water	Lake sediment	Acidic sulfide spring	Eutrophic pond

* Genome-derived.

**Table 2:** Average Nucleotide Identity (ANIb) comparison of *Rhodopseudomonas* strains. ANIb values above the species level cutoff (>95%) are marked in bold. The HC1 isolate is marked in red.

*Rps rutila* DSM 126																			
**97.6**	*Rps rutila* TIE-1																		
**97.3**	**97.3**	*Rps rutila* R1^T^																	
88.3	88.2	88.3	*Rps pantothenatexigens* JA575^T^																
88.3	88.2	88.2	**100**	*Rps thermotolerans* JA576^T^															
88.4	88.3	88.4	89.6	89.6	*Rps* sp. DX1														
88.1	88	88.1	89.3	89.3	88	*Rps* sp. XCP													
82	82.8	82.1	82.3	82.3	82.1	82.1	*Rps* sp. AAP120												
81.6	81.5	81.6	82.1	82.1	81.8	81.6	87.1	*Rps* sp. ATH 2.1.18											
81.7	81.6	81.6	82	82	81.7	81.7	86.9	87.9	*Rps infernalis* HC1										
81.8	81.7	81.8	82.3	82.4	82.3	81.8	84	83.9	83.8	*Rps* sp. HaA2									
81.2	81.1	81.2	81.6	81.6	81.3	81.2	83.3	82.9	83.1	85.6	*Rps palustris* DSM 123^T^								
81.4	81.6	81.4	81.8	81.8	81.5	81.1	83.6	83	83.1	85.7	**98.2**	*Rps palustrus* BisB5							
81	81	81.1	81.5	81.5	81.1	81.3	81.9	81.6	81.3	81.1	80.5	80.4	*Rps* sp. B29						
78.4	78.2	78.4	78.7	78.7	78.6	78.2	78.5	78.5	78.4	79.2	79.2	79.4	77.9	*Rps* sp. BisA53					
77.9	77.8	77.8	78.3	78.2	78	77.7	77.8	77.8	77.3	78.3	77.9	78.2	77.5	80.9	*Rps faecalis* JCM 11668^T^				
78.6	78.8	78.7	78.9	78.9	78.6	78.6	78.9	78.9	78.6	79.6	79.4	79.5	78.5	81.2	79.3	*Rps. rhenobacensis* DSM 12706			
78.2	78.1	78.2	78.5	78.5	78.4	77.9	78.3	78.4	78.1	79.2	79	79.2	77.9	81.4	79.3	89.3	*Rps* sp. BisB18		
78.6	78.5	78.6	79	79	78.7	78.7	79.1	78.8	78.7	80	79.4	79.7	78.2	79.5	77.8	80.5	80.7	*Rps* sp. BAL398	

## Data Availability

The *Rhodopseudomonas infernalis* HC1 Whole Genome Shotgun project has been deposited at DDBJ/ENA/GenBank under the project accession NZ_JAJNAL000000000. This version of the project (01) has the accession number NZ_JAJNAL010000000, and consists of sequences JAJNAL010000001-JAJNAL010000087.
